# Iodine‐Optimized Interface for Inorganic CsPbI_2_Br Perovskite Solar Cell to Attain High Stabilized Efficiency Exceeding 14%

**DOI:** 10.1002/advs.201801123

**Published:** 2018-10-31

**Authors:** Jingru Zhang, Zhiwen Jin, Lei Liang, Haoran Wang, Dongliang Bai, Hui Bian, Kang Wang, Qian Wang, Ningyi Yuan, Jianning Ding, Shengzhong (Frank) Liu

**Affiliations:** ^1^ Key Laboratory of Applied Surface and Colloid Chemistry Ministry of Education Shaanxi Key Laboratory for Advanced Energy Devices Shaanxi Engineering Lab for Advanced Energy Technology School of Materials Science & Engineering Shaanxi Normal University Xi'an 710119 P. R. China; ^2^ School of Physical Science and Technology & Key Laboratory for Magnetism and Magnetic Materials of MoE Lanzhou University Lanzhou 730000 China; ^3^ School of Materials Science and Engineering Jiangsu Collaborative Innovation Center of Photovoltaic Science and Engineering Jiangsu Province Cultivation Base for State Key Laboratory of Photovoltaic Science and Technology Changzhou University Changzhou Jiangsu 213164 China; ^4^ Dalian National Laboratory for Clean Energy iChEM Dalian Institute of Chemical Physics Chinese Academy of Sciences Dalian 116023 P. R. China

**Keywords:** CsPbI_2_Br, efficiency, inorganic, iodine, perovskite, solar cells

## Abstract

Recently, inorganic CsPbI_2_Br perovskite is attracting ever‐increasing attention for its outstanding optoelectronic properties and ambient phase stability. Here, an efficient CsPbI_2_Br perovskite solar cell (PSC) is developed by: 1) using a dimension‐grading heterojunction based on a quantum dots (QDs)/bulk film structure, and 2) post‐treatment of the CsPbI_2_Br QDs/film with organic iodine salt to form an ultrathin iodine‐ion–enriched perovskite layer on the top of the perovskite film. It is found that the above procedures generate proper band edge bending for improved carrier collection, resulting in effectively decreased recombination loss and improved hole extraction efficiency. Meanwhile, the organic capping layer from the iodine salt also surrounds the QDs and tunes the surface chemistry for further improved charge transport at the interface. As a result, the champion device achieves long‐term stabilized power conversion efficiency beyond 14%.

In the last few years, the organic–inorganic hybrid perovskites have attracted tremendous research due to their extraordinary optoelectronic properties, such as large absorption coefficient, high charge carrier mobility, long electron‐hole diffusion length, and tunable bandgap.^1^, ^2^, ^3^, ^4^, ^5^, ^6^ The power conversion efficiency (PCE) of the hybrid perovskite solar cells (PSCs) has reached as high as 23.3%.^7^, ^8^, ^9^, ^10^, ^11^, ^12^ Unfortunately, there are still several roadblocks preventing this promising solar cell technology from reaching commercialization; the first is the poor stability caused by its erratic organic moiety.^13^, ^14^, ^15^, ^16^, ^17^, ^18^

The general formula for lead halide perovskite is ABX_3_, where the *B* site is occupied by Pb and the *X* site by halide anions. Theoretical study concludes that the *A* site cation in the perovskite structure mainly serves to provide lattice charge compensation, and it has a trivial effect on the energy band structure.^19^, ^20^, ^21^ Moreover, there are also reports suggesting that the *A* site does not confer any fundamental advantage to the charge carrier mobilities.^22^, ^23^, ^24^, ^25^ Hence, it is desirable to replace the fragile organic group with more robust inorganic Cs cations to form more stable CsPb*X*_3_ perovskite material.^26^, ^27^, ^28^, ^29^, ^30^, ^31^ In fact, there have been studies of CsPb*X*_3_ in terms of its compositional and structural phase stability.^32^, ^33^, ^34^ Unfortunately, this Cs replacement brings about a different problem: The desired black CsPbI_3_ cubic phase (with narrow bandgap of 1.7 eV) is not the most thermodynamically stable phase at normal operational temperatures, and it may turn into the nonperovskite yellow phase quickly, even in an inert atmosphere.^35^, ^36^, ^37^, ^38^, ^39^ While halide variations would form phase‐stable CsPbBr_3_ and CsPbBr_2_I, their bandgaps are too large for photovoltaic applications.^40^, ^41^, ^42^, ^43^, ^44^ Fortunately, CsPbI_2_Br is found to have a more suitable bandgap (1.91 eV) and better thermal stability.^45^, ^46^, ^47^, ^48^

So far, there have been reports of developing highly efficient and stable CsPbI_2_Br PSCs.^49^, ^50^, ^51^ For example, Chen et al. used co‐vacuum‐deposition to prepare CsPbI_2_Br film to attain a PCE as high as 11.8%.^52^ Nam et al. used a solution‐processing method to improve the PCE to ≈10% by partially substituting Cs^+^ ions with K^+^ ions.^53^ Lau et al. boosted the PCE to 11.2% by incorporating Sr^2+^ ions into the CsPbI_2_Br.^54^ Mai and co‐workers developed a ZnO@C_60_ bilayer as the electron‐transport layer (ETL) to achieve high carrier extraction efficiency and low leakage loss. Consequently, the fabricated PSC presents PCE as high as 13.3%.^55^ In our previous work, we have designed a dimension‐profiled heterojunction structure for optimized energy alignment to decrease recombination loss during the hole‐transfer process.^56^ Such profiled structure–based PSCs resulted in a PCE of 12.39%. Further, using Mn^2+^ ions to modulate the film growth and to passivate the grain boundary defects, the champion device achieved stabilized PCE as high as 13.47%.^57^

In the present work, quantum dots (QDs) were first used to form a dimension‐graded heterojunction structure with the CsPbI_2_Br perovskite bulk film to optimize the energy alignment in the solar cell (PSC) structure.^56^ Then, we ventured to conduct interface engineering by post‐treatment of the CsPbI_2_Br perovskite film with a series of *A* site cation–based iodine salts (AI, where A = formamidinium (FA^+^), methylammonium (MA^+^), ethylenediamine (EDA^+^), phenylethylammonium (PEA^+^), or n‐butylammonium (BA^+^)) to achieve further improved device performance. It is found that such process leads to decreased recombination loss and further improved hole extraction efficiency. Consequently, we successfully demonstrated a highly efficient planar heterojunction PSC with a record PCE as high as 14.12% for this type of device.

**Figure** **1**a illustrates the preparation process of the CsPbI_2_Br light absorption layer for the PSCs, wherein the CsPbI_2_Br film was fabricated by one‐step spin‐coating, with an ultrathin CsPbI_2_Br QDs layer deposited onto the CsPbI_2_Br film. Then the prepared film was post‐treated by soaking in the formamidinium iodide (FAI) ethyl acetate (EA) solution for different periods of time to passivate the QDs surface and grain boundary defects. The iodine‐ion–enriched CsPbX_3_ layer formed on the top of CsPbI_2_Br film is expected to modify the film surface for proper band edge bending. Meanwhile, the FA^+^ may modify the CsPbI_2_Br surface by passivating the grain boundaries and surface defects, resulting in effectively decreased recombination loss, improved charge transport at the interface, and increased hole extraction efficiency.

**Figure 1:**
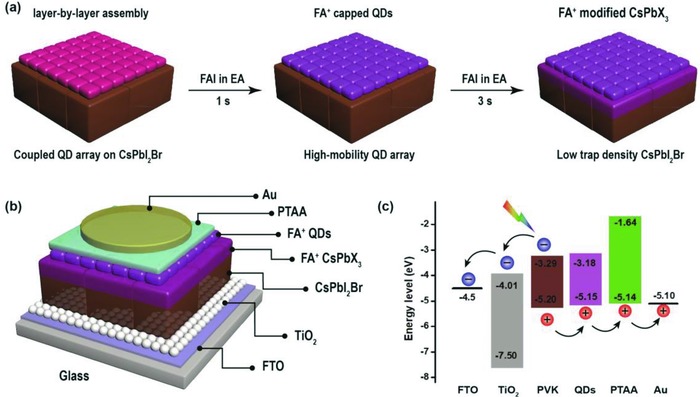
a) Schematic diagram of the CsPbI_2_Br QDs/film with FAI post‐treatment. b) Schematic device structure. c) Energy‐level diagram.

Figure 1b shows the designed final CsPbI_2_Br PSC, consisting of fluorine‐doped tin oxide (FTO) glass/TiO_2_/CsPbI_2_Br/FA^+^CsPbX_3_/FA^+^QDs/poly[bis(4‐phenyl) (2,4,6‐trimethylphenyl)amine] (PTAA)/Au, wherein the TiO_2_ film deposited on the FTO glass is employed as the ETL, the CsPbI_2_Br film fabricated thereupon as the active absorbing layer, the FA^+^CsPbX_3_ to passivate the grain boundaries and to adjust the band edge bending, the FA^+^QDs to form a graded structure, the PTAA film as the hole‐transport layer (HTL), and the Au coating as the top anode. Figure 1c illustrates the energy level alignment for each layer in the structure of the PSCs. The energy levels of each layer were measured as reported in our previous paper^56^: The valence band (VB) was obtained using ultraviolet photoelectron spectroscopy (UPS) spectrum while the conduction band (CB) based on the optical absorption spectra. Clearly, under illumination, the Fermi levels of the film balance at the same level due to the electron and hole flows in opposite directions reaching the lowest CB and highest VB, respectively.^56^ For such designed graded energy level, a reduction of the work function (WF) and a downward vacuum level shift at the interface are produced. Meanwhile, an additional driving force for holes is generated owing to the built‐in electric field of the VB offset. Moreover, the CB offset offers an energy barrier that restrains photogenerated electrons from flowing to the counter electrode.^56^

Scanning electron microscopy (SEM, **Figure** **2**) and atomic force microscope (AFM; Figure S1, Supporting Information) images were used to study the surface morphology as a function of FAI treatment time for two separate types of films: CsPbI_2_Br‐FA and CsPbI_2_Br‐QDs‐FA. It shows that in both categories, the pristine CsPbI_2_Br film without any FAI post‐treatment shows the smoothest surface morphology (Figure 2a). The surface of the CsPbI_2_Br films become more roughened as the FAI treatment prolongs (Figure 2b–d and Figure S1, Supporting Information). This should be caused by halide exchange while in the EA solution, the Br^−^ in the CsPbI_2_Br film is exchanged with I^−^ from FAI in the EA solution.^58^, ^59^ Meanwhile, with greater FAI processing time, the water contact angle is increased from 47.8° to ≈61.4°. Such phenomenon indicates that the FA^+^ (with its hydrophobic terminal groups) was modified on the surface of the CsPbI_2_Br film. Simultaneously, we compared SEM and AFM images of CsPbI_2_Br films soaked in EA solutions containing different AI salts for 3 s. It was obvious that rougher surface also formed when the processing time reached 3 s (see in Figure S2, Supporting Information). Hence, we speculated that there will be an ion exchange reaction between the AI and the CsPbI_2_Br film on the surface to form A^+^CsPbX_3_. When we used CsPbI_2_Br QDs/film post‐treated with FAI (Figure 2e–h), the surface also turned rough, and the water contact angle was obviously increased for the induced CsPbI_2_Br QDs. Figure S3 in the Supporting Information shows the characteristics of the synthesized QDs. From the Fourier transform infrared (FTIR) spectroscopy (Figure S4, Supporting Information), we found a strong chemical interaction between FA^+^ and the QDs after the FAI post‐treatment. It is believed the AI capping surrounding the QDs could tune the layer's surface chemistry resulting in a high‐quality QDs layer and improve the charge transport.^36^

**Figure 2:**
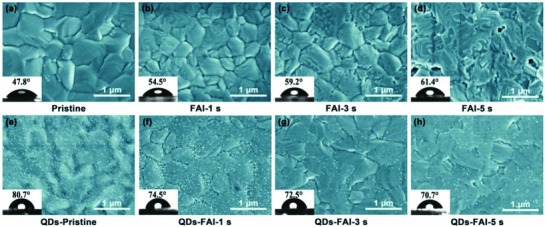
SEM images of the a–d) CsPbI_2_Br films and e–h) CsPbI_2_Br QDs/films soaked in FAI EA solution for different times. The insets are the results of water droplet contact angle measurements.

To confirm whether the post‐treatment with FAI is useful, we conducted X‐ray diffraction (XRD) and photoelectric performance characterizations. As shown in **Figure** **3**a, the series of XRD patterns indicates that there is no obvious change in the XRD peaks for different treatment times. This indicates that the lattice constant of the whole CsPbI_2_Br crystal has not changed, and neither FA^+^ nor I^−^ are incorporated into the original crystal; only an ultrathin I‐enriched FA^+^CsPbX_3_ layer formed on the surface of the film. Optical absorption (Figure 3b) and photoluminescence (PL, Figure 3c) measurements were also carried out on the CsPbI_2_Br films with different FAI processing times; with increasing FAI processing time, the positions of the CsPbI_2_Br film absorption edge and PL peak have a slight redshift caused by the induced ultrathin I‐enriched FA^+^CsPbX_3_ layer. It has been proven that the PL peak undergoes an obvious redshift when the I^−^ anion content is increased in CsPbX_3_ (Figure S5, Supporting Information). Specially, the much‐enhanced PL intensity should be caused by the FA^+^ modified CsPbI_2_Br surface and passivated surface defects.

**Figure 3:**
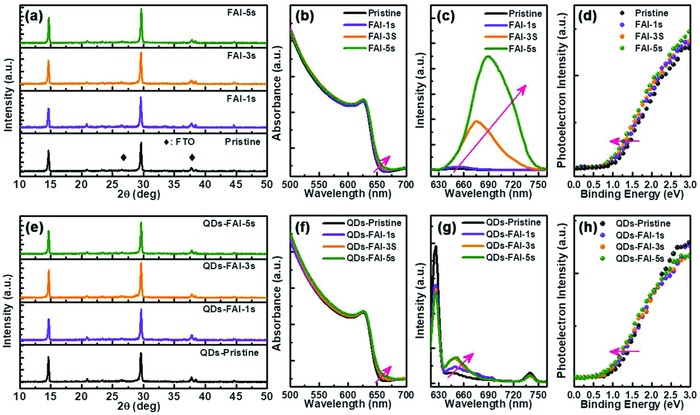
Performance comparison of the a–d) CsPbI_2_Br films and e–h) CsPbI_2_Br QDs/films soaked in FAI EA solution for different times: a,d) XRD patterns; b,f) absorption spectra; c,g) PL spectra; d,h) VB XPS spectra.

Figure 3e shows the XRD patterns of CsPbI_2_Br QDs/films that underwent the FAI post‐treatment. The XRD patterns show no obvious change compared with those of the pristine CsPbI_2_Br film. The absorption edge and PL peak also have a slight redshift with increasing processing time. This resulted mainly from the formed FA^+^ QDs and FA^+^CsPbX_3_.^59^ Figure S6 in the Supporting Information is a comparison of the XRD and absorption patterns of the CsPbI_2_Br films soaked for 3 s in EA solutions containing different AI salts, and the conclusion is the same as for the FAI treatment.

To characterize the interface and band alignment around the graded heterojunction, VB X‐ray photoelectron spectroscopy (XPS) spectra (Figure 3d,h) were measured. The VB energy can be derived from the intersection of the linear portion of the spectra near the Fermi edge. All the VBs are shifted upwards when the CsPbI_2_Br films were soaked in FAI (Table S1, Supporting Information). This result supports our hypothesis that the surface treatment by iodine would shift the VB of the active layer upwards at the QDs/film interface. It is expected that improved energy alignment will render improved carrier collection.^60^

For the purpose of identifying the effects of the FAI treatment on the films, XPS measurements were further investigated. **Figure** **4**a shows the XPS spectra for each element at the surface. After FAI treatment, N was clearly observed. Figure 4b shows the I/Pb, Br/Pb, N/Pb, and I/Br atomic ratios at the surface of the CsPbI_2_Br films. From the XPS results, we find that the N/Pb and I/Br atomic ratios each has an evident increase with longer FAI processing time. This is mainly because FA^+^CsPbX_3_ formed on the surface of the CsPbI_2_Br films after the FAI treatment, which matches the absorption and PL results. As a result of the FAI treatment, the Cs 3d5, Pb 4f, I 3d5, and Br 3d peaks shift to higher binding energy, indicating that the chemical structures of the surface have been modified (see Figure 4c–f).

**Figure 4:**
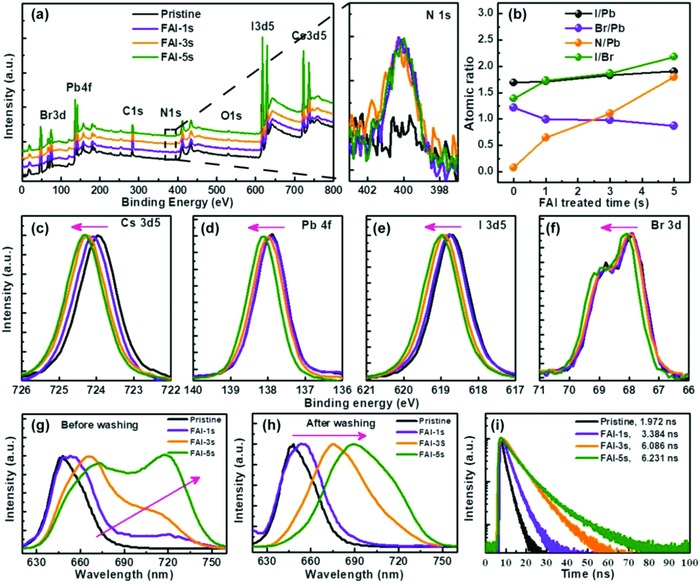
Comparison of the characteristics of the CsPbI_2_Br films soaked in FAI EA solution for different times: a) XPS spectra; b) atomic ratio evolution of I/Pb, Br/Pb, N/Pb, and I/Br; c–f) XPS spectra for Cs 3d5, Pb 4f, Br 3d, and I 3d5; g) PL spectra for the films before EA washing for different soaking times; h) PL spectra of the films after EA washing for different soaking times; i) decay curves.

To clarify the exchange process, we measured the PL spectra before and after neat EA treatment (Figure 4g,h). The PL spectra show that, before EA cleaning, there is a strong PL peak at 720 nm that increases in intensity with increasing FAI treatment time. This peak should be from the FACsPbI_x_Br_y_ formed through FAI alloying with the CsPbI_2_Br as shown in Figure S4 in the Supporting Information. After EA washing, there is mainly one PL peak, and the position of the PL peak has an evident redshift with increasing FAI processing time. This phenomenon indicates that a thin layer of FA‐ and I‐enriched CsPbI_2_Br film is formed. The time‐resolved PL decay profiles were measured for the CsPbI_2_Br/FTO films with different FAI treatment times, as shown in Figure 4i. It is clear that the longer the treatment time, the larger the charge carrier lifetime. The same trend has also been observed in the CsPbBrI_2_/QDs film (Figure S7, Supporting Information), indicating that the FAI treatment effectively passivates surface defects, resulting in effectively decreased recombination loss with improved hole extraction efficiency.

To confirm the effectiveness of the FAI post‐treatment on device performance, the corresponding films were used as the photoactive layer in PSCs for photovoltaic performance evaluation. The current density–voltage (*J*–*V*) curves, the corresponding external quantum efficiency (EQE) curves, and photovoltaic parameters are presented in Figure S8 in the Supporting Information and **Table** **1**. The reference PSC had inferior performance with a PCE of 12.48%, an open‐circuit voltage (*V*_OC_) of 1.190 V, a short‐circuit current density (*J*_SC_) of 14.11 mA cm^−2^, and a fill factor (*FF*) of 74.3%. Clearly, even for a 1s FAI treatment, all of the device performance metrics increased markedly. The device performance is improved significantly to 12.95%. For a 3s FAI treatment, the PCE reached a maximum of 13.23%. The improvement probably resulted mainly from FA^+^ passivating grain boundary and surface defects. With further increased treatment time, the device performance dropped quickly, which was likely caused by the formation of an amorphous FA^+^CsPbX_3_ layer that is too thick. To verify the performance of AI in devices, we compared the performance of PSCs based on CsPbI_2_Br films soaked for 3 s in EA solutions containing different AI salts and found that different AI salts can also improve the performance of the device to a certain extent. While the FAI was found to be the best post‐treatment salt, we believe this is because the FA^+^‐modified film had the highest carrier mobility,^36^ as reported. Finally, for the PSCs based on CsPbI_2_Br QDs/film, the PCE reached 14.12% when the absorbing layer was treated with FAI for 3 s because as mentioned above, the QDs largely induced proper band edge bending, and the FA^+^ capping surrounded the QDs to yield a high‐quality QDs layer.

**Table 1:** Parameters of the PSCs based on different CsPbI_2_Br films extracted from Figure S6 in the Supporting Information

Perovskite film	Halide salt	Treat time [s]	*J*_SC_ [mA cm^−2^]	*V*_OC_ [V]	*FF* [%]	PCE [%]	*J*_SC_ [mA cm^−2^]
CsPbl_2_Br	FAI	0	14.11	1.190	74.3	12.48	13.86
		1	14.44	1.191	75.3	12.95	14.16
		3	14.48	1.201	76.1	13.23	14.27
		5	14.61	1.156	75.8	12.80	14.35
CsPbl2Br/QDs	FAI	0	14.35	1.215	74.5	12.99	14.14
		1	14.46	1.220	77.2	13.62	14.15
		3	14.51	1.223	79.6	14.12	14.33
		5	14.69	1.205	77.4	13.70	14.49
CsPbbBr	MAI	3	14.20	1.205	75.1	12.85	13.89
	EDAl_2_	3	14.24	1.200	75.7	12.94	14.01
	BAI	3	14.28	1.212	74.4	12.88	12.93
	PEAI	3	14.32	1.207	75.3	13.01	14.08

Shown in **Figure** **5**a is the typical *J*–*V* curves of the champion performing device measured using both the reverse and forward scan directions, with the key parameters summarized in the inset. It should be noted that the device shows very little hysteresis. Such phenomenon, believed to be caused by the effect of light‐enhanced ion migration in hybrid perovskite, is eliminated in the all‐inorganic Cs‐substitution system.^29^ The EQE spectrum of the champion cell is shown in Figure 5b, where the integral current density as a function of wavelength is also presented. The vast majority of the EQE values exceed 80% in the absorbance range from 350 to 650 nm. To investigate the steady power output (SPO) and steady‐state current performance, we applied 1.08 V to the solar cells under continuous AM 1.5 simulated sun light. Figure 5c shows that the SPO and *J*_SC_ remain stable at ≈14% and ≈13 mA cm^−2^, respectively, during the entire period of 120 s measurement.

**Figure 5:**
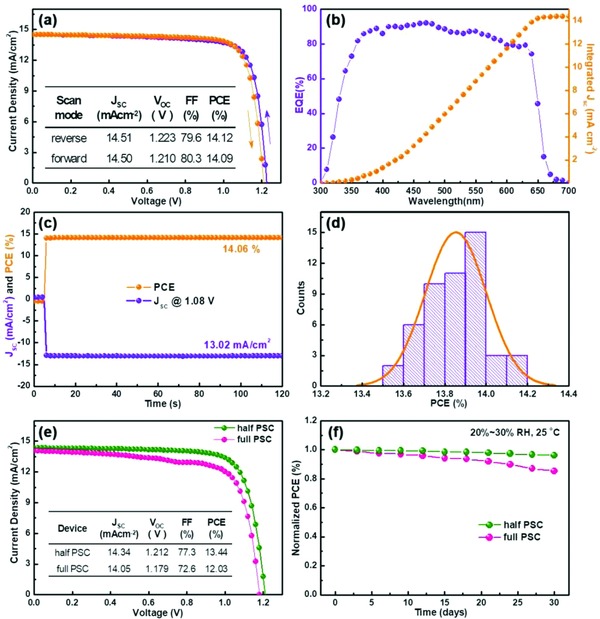
Performance for the champion device: a) *J*–*V* characteristics under both the reverse and forward scan directions; b) EQE and the integrated product of the EQE curve with the AM1.5G photon flux; c) PCE and *J* measured as a function of time for the cells biased at 1.08 V; d) PCE distribution histogram of 50 devices; e) *J*–*V* characteristics of the best‐performance half and full PSCs without any encapsulation after storage for one month (25 °C and RH = 25–35%); f) Long‐term stability of the best‐performing device stored without encapsulation (25 °C and RH = 25–35%).

To better measure the performance of the devices, we generated the PCE distribution histogram of 50 devices, of which the average PCE was ≈13.85% (Figure 5d). This represents the highest average efficiency achieved yet for fully inorganic lead halide perovskites. It is known that the phase transition temperature for the orthorhombic to cubic perovskite transition decreases with increasing bromide content. The performance and stability for this mixed halide composition, CsPbI_2_Br, are shown in Figure 5e,f. The bare fabricated perovskite film (half PSC) and the corresponding device (full PSC) without any encapsulation were both stored and tested upon exposure to the ambient environment (in air at relative humidity of 25–35% and 25 °C). The experimental results show that the performance of the half device is better than the full; this observation supports our speculation that by replacing the HTL with other materials, e.g., metal oxides or carbon electrodes, the stability of the full cells could be further improved.

In summary, the AI treatment developed here provides a general method for optimizing the interfacial properties of lead halide PSCs. In the applied QDs/film structure, an ultrathin iodine‐ion–enriched perovskite layer was formed on the top of the CsPbI_2_Br film, and the QDs surfaces were proven to be capped with AI after AI salt post‐treatment. We found that such a phenomenon leads to proper band edge bending, decreased surface defects, and a high‐quality modified QDs layer. As a consequence, these changes proved effective at decreasing recombination loss and improving hole extraction efficiency. More specifically, the FAI‐treated device yields an ultrahigh PCE of 14.14%, which positioning is greater than above the best reported PCE for CsPbI_2_Br PSCs to date. We believe that this strategy should have significant potential for future applications in other optoelectronic devices.

## Conflict of Interest

The authors declare no conflict of interest.

## Supporting information

Supplementary
