# Supplementary material for: Iodine‐Optimized Interface for Inorganic CsPbI2Br Perovskite Solar Cell to Attain High Stabilized Efficiency Exceeding 14%
Source: Adv Sci (Weinh). 2018 Oct 31;5(12):1801123. doi: 10.1002/advs.201801123 (PMC6299820; doi:10.1002/advs.201801123)
Supplement: Supplementary file 1 — Supplementary [file ADVS-5-1801123-s001.pdf]

Copyright WILEY-VCH Verlag GmbH & Co. KGaA, 69469 Weinheim, Germany, 2018.

ADVANCED SCIENCE
Open Access

Supporting Information

for *Adv. Sci.,* DOI: 10.1002/advs.201801123

Iodine-Optimized Interface for Inorganic $CsPbI_2Br$ Perovskite Solar Cell to Attain High Stabilized Efficiency Exceeding 14%

Jingru Zhang, Zhiwen Jin, Lei Liang, Haoran Wang, Dongliang Bai, Hui Bian, Kang Wang, Qian Wang,* Ningyi Yuan, Jianning Ding, and Shengzhong (Frank) Liu**

Supporting Information

Iodine Optimized Interface for Inorganic $CsPbI_2Br$ Perovskite Solar Cell to Attain High Stabilized Efficiency exceeding 14%

Jingru Zhang[1], Zhiwen Jin[1,2,*], Lei Liang[1], Haoran Wang[1], Dongliang Bai[1], Hui Bian[1], Kang Wang[1], Qian Wang[1,2,*], and Shengzhong (Frank) Liu[1,3,*]

[1]Key Laboratory of Applied Surface and Colloid Chemistry, Ministry of Education; Shaanxi Key Laboratory for Advanced Energy Devices; Shaanxi Engineering Lab for Advanced Energy Technology; School of Materials Science & Engineering, Shaanxi Normal University, Xi'an, 710119, P. R. China.
E-mail: jinzhiwen@snnu.edu.cn, wangqian16@snnu.edu.cn

[2]School of Physical Science and Technology & Key Laboratory for Magnetism and Magnetic Materials of MoE, Lanzhou University, Lanzhou 730000, China

[3]Dalian National Laboratory for Clean Energy; iChEM, Dalian Institute of Chemical Physics, Chinese Academy of Sciences, Dalian, 116023, P. R. China
E-mail: szliu@dicp.ac.cn

Keywords: iodine, $CsPbI_2Br$, inorganic, perovskite, solar cell, efficiency

Experimental Section

Solution Preparation Section:

Materials Preparation: All of the materials used in this study were purchased from Sigma-Aldrich and were used as received without further purification. 500-nm FTO-coated glass was used as the substrates.

Precursor Solution Preparation: $CsPbI_2Br$ precursor solution was prepared through dissolving $CsI:PbBr_2:PbI_2$ (1:0.5:0.5) in DMF at 0.6 M in a N_2 glovebox and stirring at 55 ℃ for 2 h.

$CsPbI_2Br$ QDs Solution Preparation: $CsPbI_2Br$ QDs were synthesized as in our previous report.[1,2] The prepared $CsPbI_2Br$ QDs were dispersed in octane at 10 mg/ml.

Saturated AI precursor solution Preparation: As an ionic salt, the solubility of AI salts in the non-polar solvent used is very small. Therefore, the AI concentration in the liquid phase at any given time is controlled by its limited solubility. The mixture was actively stirred at room temperature for 30 minutes. Upon centrifugation to remove excess salt, the resultant solution appears to be clear.

HTL Solution Preparation: A solution was prepared by dissolving PTAA (90 mg), a sulfonyl imide (Li-TFSI, 22 μL) solution (520 mg Li-TFSI in 1 mL acetonitrile) and tert-butylpyridine (TBP, 36 μL) in 1 mL of CB solution.

All used solutions were filtered through a 0.4-μm-pore PTFE filter and stored in a dry nitrogen atmosphere.

Device Fabrication Section:

Preparation of TiO_2 Blocking Layer: A 25 × 25 mm^2 piece of FTO-coated glass was washed sequentially with detergent, deionized water, acetone, and isopropanol with ultrasonication for 10 min each, and then was dried by N_2 and treated in an O_2 plasma. The clean substrate was immersed in a 40 mM $TiCl_4$ aqueous solution for 30 min at 70 ℃ and washed with distilled water and ethanol, followed by annealing at 200 ℃ for 30 min in air to form a compact n-type blocking layer of TiO_2.

Growth of the $CsPbI_2Br$ Film: The $CsPbI_2Br$ precursor solution was spin-coated onto the substrate at 4000 rpm for 30 s, and immediately after, the films were sintered at 300 ℃ for 10 min. Then, the $CsPbI_2Br$ QDs solution was dropped onto the prepared film, and spin-dried at 2000 rpm for 30 s with the film quickly dipped in neat ethyl acetate solution and dried with a stream of air. Finally, the films were post-treated by soaking the film in AX EA solution for different times before rinsing with EA. The best-performance device is based on the FAI solution treatment of the $CsPbI_2Br$/QDs film for 3s.

Assembly of the Solar Cells: An HTL film was prepared by spin-coating the HTL solution onto the above $CsPbI_2Br$ film at 4000 rpm for 30 s. Finally, a gold electrode

with a thickness of ~70 nm was thermally evaporated onto the HTL-coated film to finish the device fabrication.

Device Storage: The bare fabricated perovskite film (half PSC) and the corresponding devices (full PSC) without any encapsulation were both stored and tested upon exposure to the ambient environment (in air at relative humidity of 25%~35% and 25 ℃). For measurement of the electrical properties of the half PSC, the HTM and Au electrode were fabricated.

Characterization Section:

Thin Film Characterization: The film surface morphology was characterized by FESEM (SEM, Jeol SU-8020). The TEM images were obtained using a FEI Tecnai T20 equipped with a Gatan SC200 CCD camera and LaB6 filament operated at 200 kV. Contact angles were measured on an OCA20 machine. XRD patterns of the samples were obtained using a Bruker D8 GADDS Diffractometer using the Cu Kα line. FTIR spectra were measured on a Bruker EQUINX55 spectrometer with 128 scans at a resolution of 2 cm^{-1}, and the spectra were recorded in dry air with KBr as the background. The XPS measurements were performed in a VG ESCALAB MK2 system with monochromatized Al Kα radiation at a pressure of 5.0×10^{-7} Pa. The UPS measurements were analyzed using a Thermo Scientific ESCA Lab 250Xi system with helium gas admitted employing the HeI (21.22 eV) emission line. The UPS measurements were analyzed using a Thermo Scientific ESCA Lab 250Xi system with helium gas admitted employing the HeI (21.22 eV) emission line. The optical absorbance spectra were collected using a Shimadzu UV-3600 double beam spectrometer. Absorbance spectra were collected using a Shimadzu UV-3600 double beam spectrometer using the slowest scanning rate, with 1 s integration and a 2 nm slit width. PL spectra were measured using an HORIBA Jobin Yvon Fluoro Log2 spectrofluorometer. TRPL spectra were acquired according to a time-correlated single photon counting method using an Edinburgh Instruments FluoTime 300 fluorescence spectrometer.

Device Measurement: J-V curves were measured at 25 ℃ under AM1.5G (100 mW/cm^2) illumination (reverse (from V_{OC} to I_{SC}) scan mode with a scan rate of 30 mV/s). A black cardboard mask with a window area of 0.09 cm^2 was clipped onto the glass side to define the active area of the cell. The spectral response was taken by an EQE measurement system (QEX10, PV Measurement), which was equipped with a monochromator, a lock-in amplifier, a Xe lamp, and a current-voltage amplifier. Prior to the use of the light, the spectral response and the light intensity were calibrated using a mono-silicon detector.

References:

[1] Q. Wang, Z. Jin, D. Chen, D. Bai, H. Bian, J. Sun, G. Zhu, G. Wang and S. Liu, *Adv. Energy Mater.* **2018**, 1800007.

[2] J. Zhang, D. Bai, Z. Jin, H. Bian, K. Wang, J. Sun, Q. Wang and S. Liu, *Adv. Energy Mater.* **2018**, 1703246.

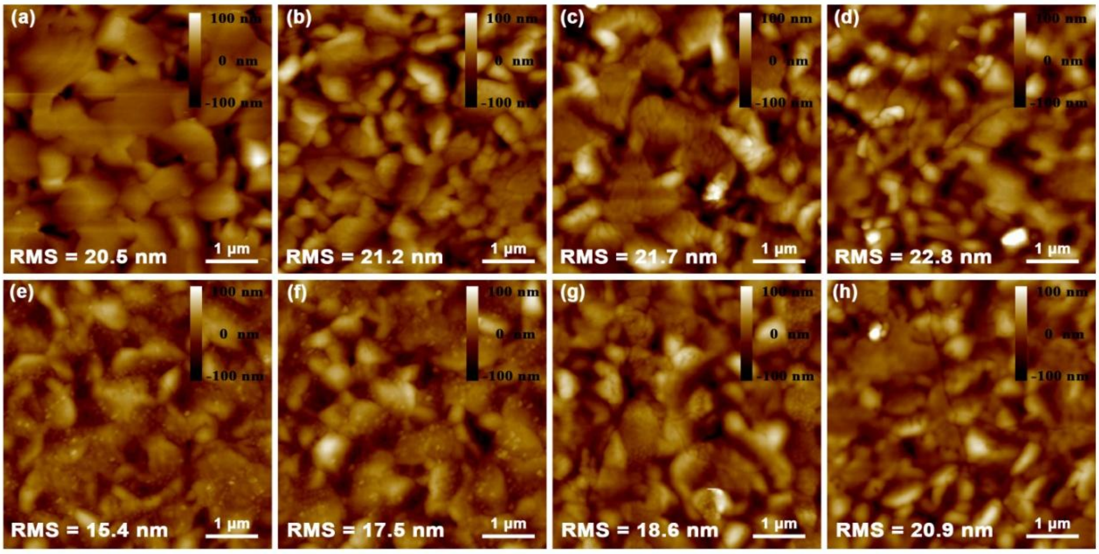

Figure S1. AFM images of the (a-d) $CsPbI_2Br$ films and (e-h) $CsPbI_2Br$ QDs/films soaked in FAI EA solution for different times.

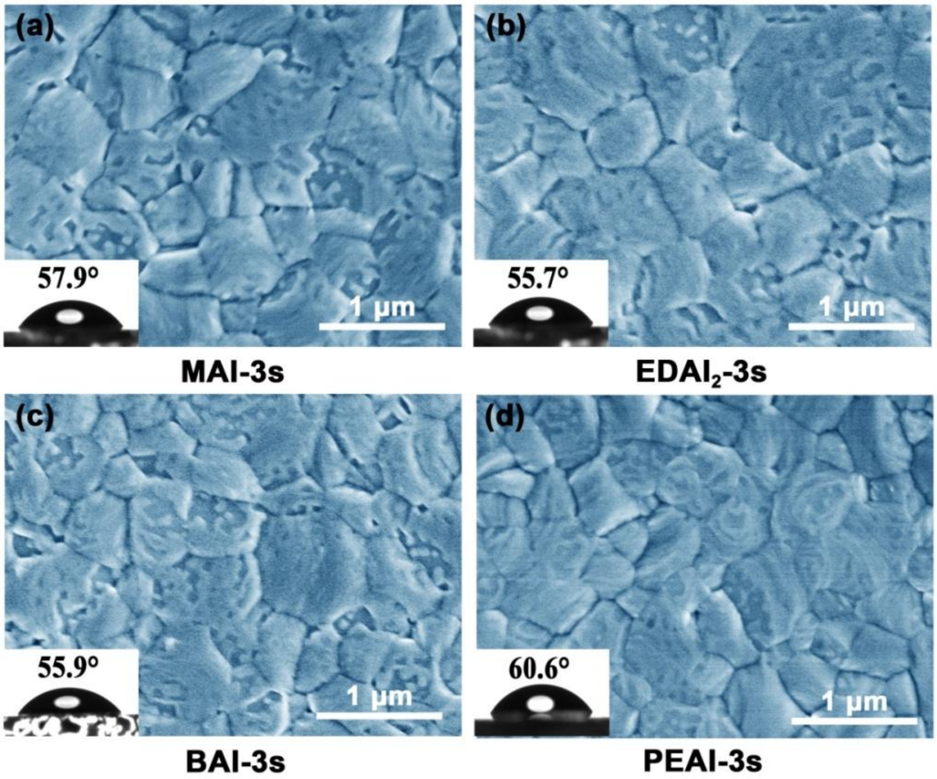

Figure S2. SEM images of the $CsPbI_2Br$ films soaked for 3 s in EA solutions containing different AI salts: (a) for MAI; (b) for $EDAI_2$; (c) for BAI; (d) for PEAI. The insets are the results of water droplet contact angle measurements.

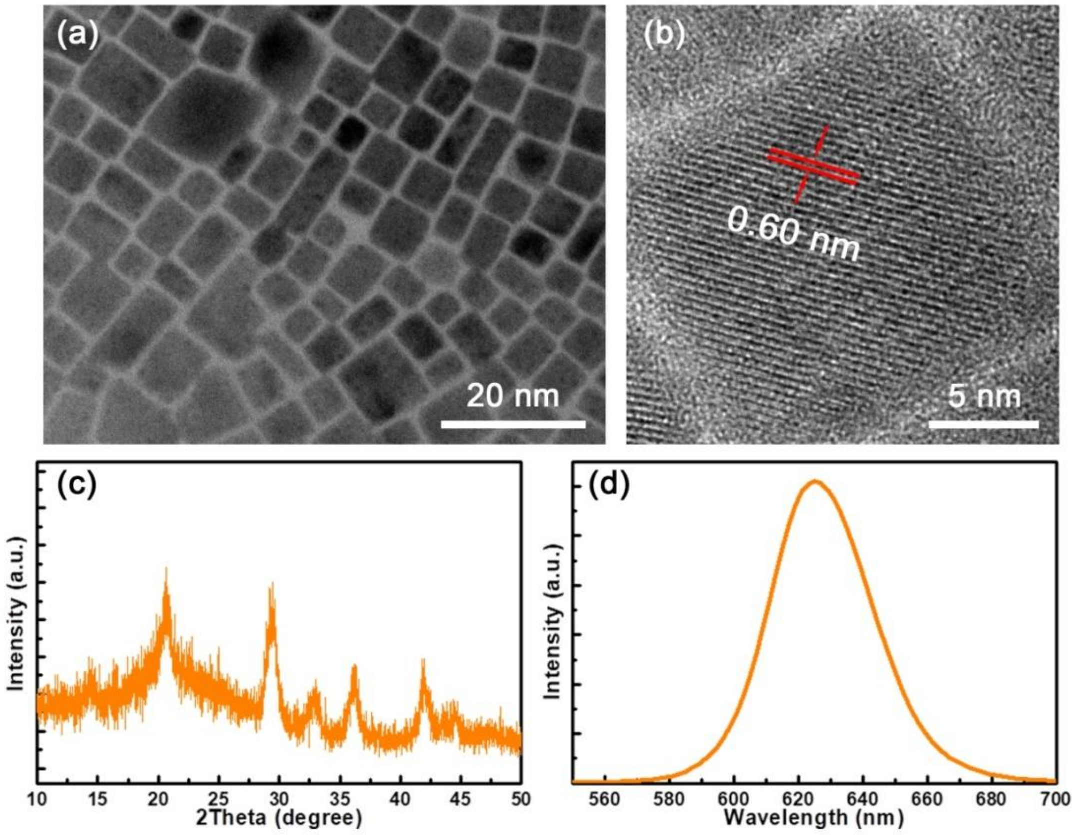

Figure S3. (a) TEM image of the $CsPbI_2Br$ QDs; (b) HRTEM image of $CsPbI_2Br$ QDs; (c) XRD pattern; (d) PL spectrum.

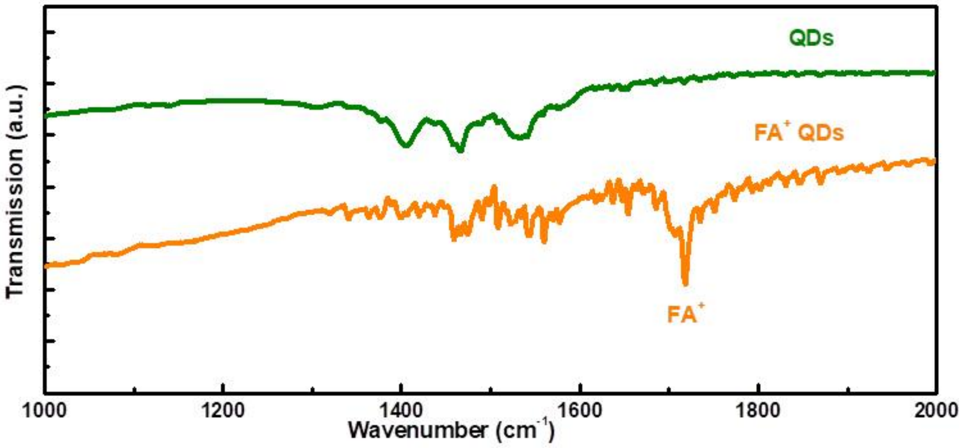

Figure S4. FTIR spectra of $CsPbI_2Br$ QDs with and without FAI post-treatment.

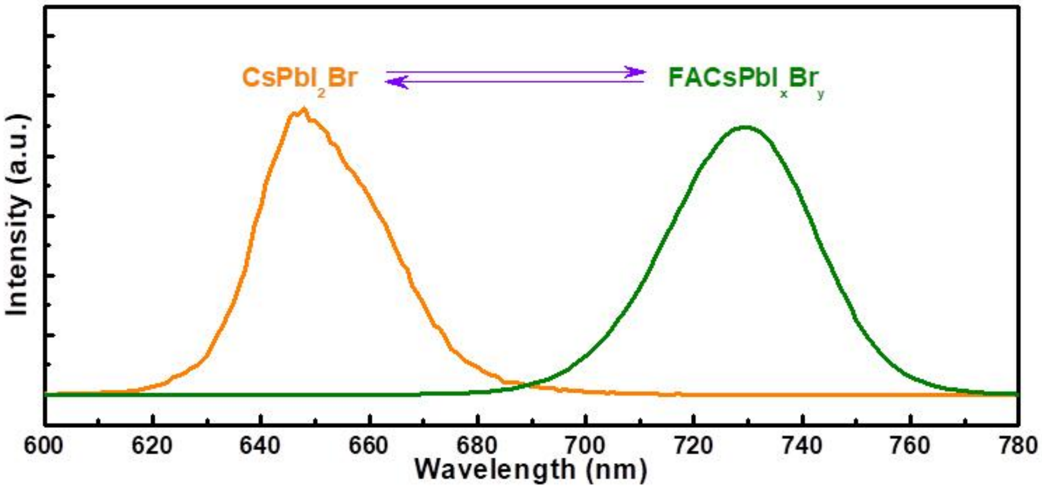

Figure S5. PL spectra of the $CsPbI_2Br$ film and the $FACsPbI_xBr_y$ film.

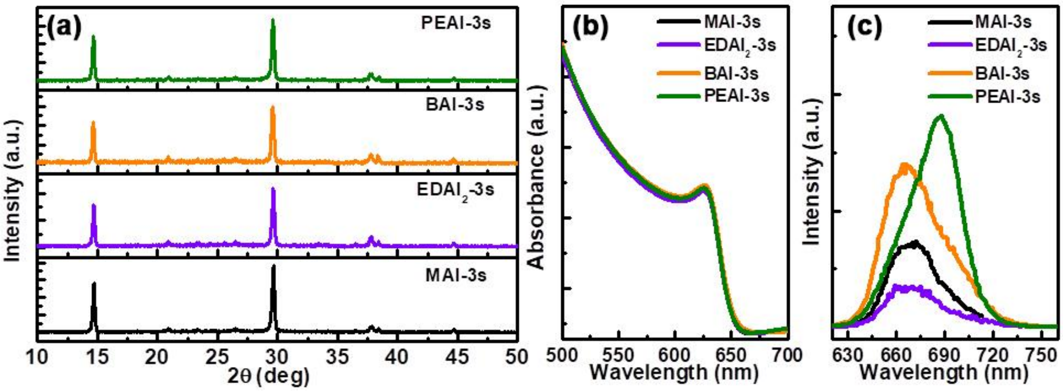

Figure S6. Comparison of the characteristics of the $CsPbI_2Br$ films soaked in EA solutions containing different AI salts: (a) XRD patterns; (b) absorption spectra; (c) PL spectra.

Table S1. VB data of different $CsPbI_2Br$ films extracted from **Figure 3d** and **Figure 3h**.

Perovskite film	$CsPbI_2Br$				$CsPbI_2Br$/QDs			
FAI treat time (s)	0	1	3	5	0	1	3	5
VB (eV)	1.107	1.049	0.982	0.939	0.983	0.932	0.860	0.826

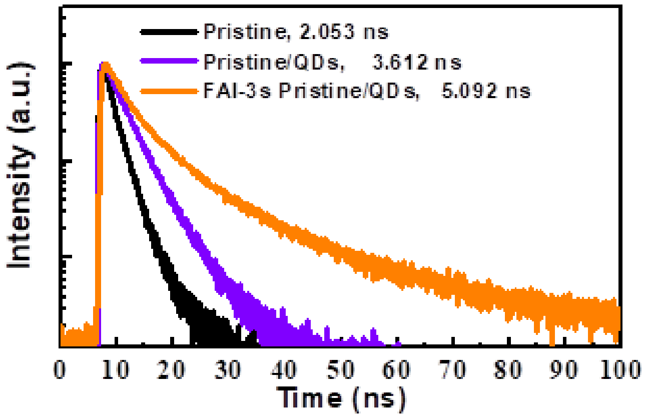

Figure S7. Comparison of the TRPL for the $CsPbI_2Br$ thin films, with QDs and also with FA.

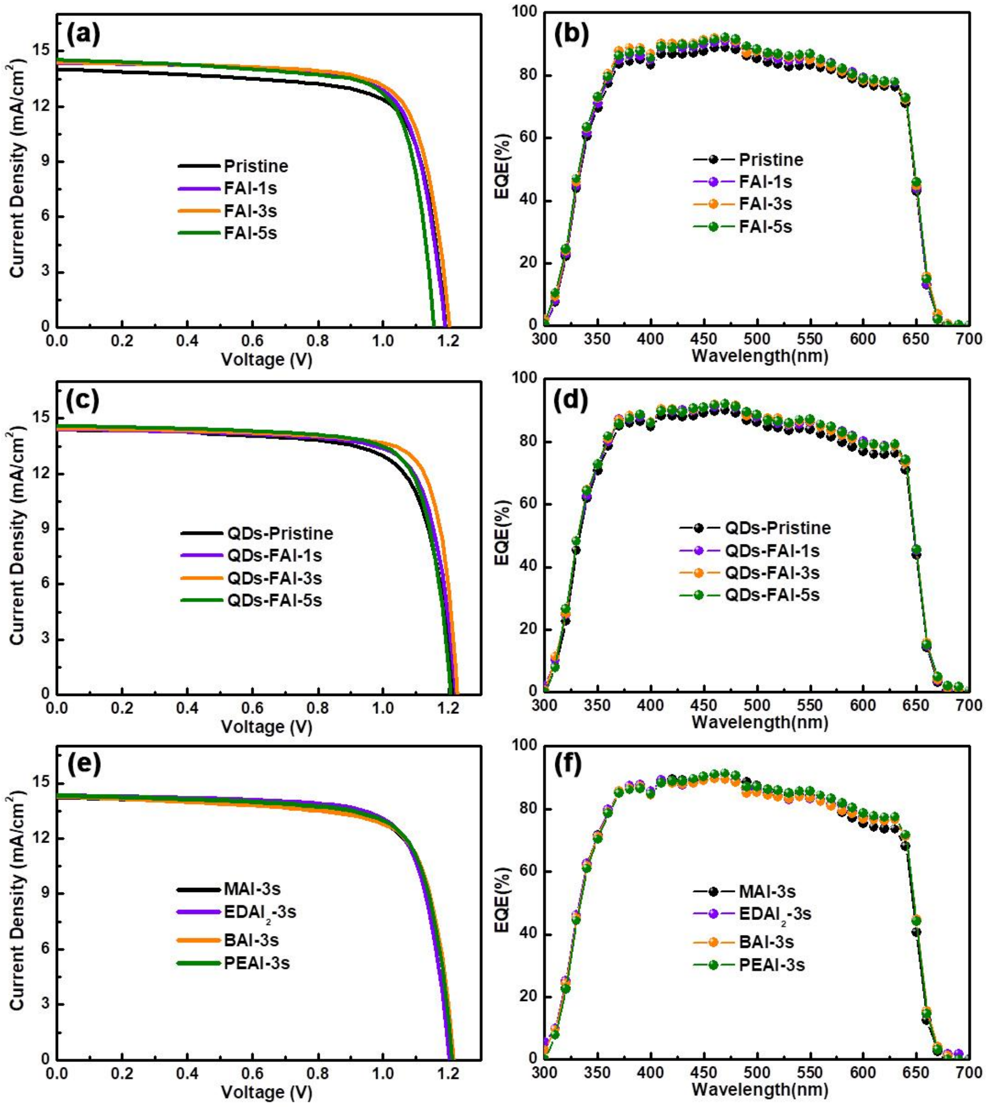

Figure S8. Comparison of the related *J-V* characteristics and EQEs: (a) and (b) $CsPbI_2Br$ films soaked in FAI EA solution for different times; (c) and (d) $CsPbI_2Br$ QDs/films soaked in FAI EA solution for different times; (e) and (f) $CsPbI_2Br$ films soaked for 3 s in EA solutions containing different AI salts.
